# The role of irradiation in the management of the axilla in early breast cancer patients

**DOI:** 10.3389/fonc.2023.1151460

**Published:** 2023-06-26

**Authors:** Thiraviyam Elumalai, Urvashi Jain, Charlotte E. Coles, John R. Benson

**Affiliations:** ^1^ Cambridge Breast Unit, Cambridge University Hospitals NHS Foundation Trust, Cambridge, Cambiridge, United Kingdom; ^2^ Department of Oncology, University of Cambridge, Cambiridge, United Kingdom; ^3^ School of Medicine, Anglia Ruskin University, Cambridge and Chelmsford, Cambiridge, United Kingdom

**Keywords:** axillary radiotherapy, breast cancer, sentinal lymph node biopsy, regional nodal irradiation (RNI), neoadjuvant chemotherapy

## Abstract

The need for axillary radiotherapy in patients with invasive breast cancer (IBC) has been a topic of great debate in the last decade. Management of the axilla has evolved significantly over the past four decades with a trend towards de-escalation of surgical interventions and the aim of reducing morbidity and enhancing QOL without compromising long-term oncology outcomes. This review article will address the role of axillary irradiation with a focus on the omission of completion axillary lymph node dissection in selected patients with sentinel lymph node (SLN) positive early breast cancer (EBC) with reference to current guidelines based on evidence to date.

## Introduction

As advances in breast cancer treatment have led to improved survival, there is a greater focus on the impact of treatment on quality of life (QOL) in the context of survivorship. Management of the axilla has evolved significantly over the past four decades with a trend towards de-escalation of surgical interventions and the aim of reducing morbidity and enhancing QOL without compromising long-term oncology outcomes. The introduction of systemic treatments has further facilitated this approach of de-escalation. A principal motivation for seeking alternatives to axillary lymph node dissection (ALND) has been to reduce rates of lymphoedema and other upper limb morbidities associated with standard forms of ALND. Lymphoedema can be severe and impair QOL substantially for patients following breast cancer treatment, both in terms of symptoms and loss of function. There has been extensive research done over the past few years to optimise axillary management and this continues to evolve (1–4). This article will address the role of axillary irradiation with a focus on omission of completion ALND in selected patients with sentinel lymph node (SLN) positive early breast cancer (EBC) with reference to current guidelines based on evidence to-date.

### Evidence for axillary RT replacing ALND in sentinel node positive axilla

There are two trials (AMAROS and OTOASOR) that have directly compared axillary radiotherapy (RT) to ALND in SLN positive EBC patients and these are discussed in more detail below.

The AMAROS trial was led by the European Organisation for Research and Treatment of Cancer (EORTC) group from 2001-2010 and included 1425 patients with clinically node negative breast cancer at presentation but a subsequent positive SLN biopsy. Patients were randomised to either ALND (anatomical levels 1, 2 & 3, including at least ten nodes) or axillary RT (50Gy in 25 fractions over five weeks, including all axillary levels and medial supraclavicular fossa) (5). Of note, randomisation was performed prior to SLN biopsy, which minimised selection bias. After exclusions were applied, 744 and 681 patients in the ALND and axillary RT arms, respectively were included in the per-protocol and intention-to-treat analyses. Patient characteristics are especially important in applying the results of this trial to routine clinical practice and are highlighted in **Table 1**.

**Table 1:** Patient summary characteristics- AMAROS trial.

40% premenopausal	
48% grade 2 cancer	
82% had BCS	
95% with 1-2 positive SLN	
60% with macro metastases	
90% received further systemic treatment	
61% received adjuvant chemotherapy	
78% received endocrine treatment	

Within the ALND group, one-third of patients (220/672) had additional positive non-sentinel lymph nodes.

The primary intent of the AMAROS trial was to confirm non-inferiority of axillary RT compared with ALND with respect to 5-year axillary recurrence in patients with positive SLN(s). On the assumption of a 2% 5-year axillary recurrence rate in the ALND group, non-inferiority was defined as axillary recurrence no higher than 4% in the axillary RT group. A total of 52 events were required to ensure a power of 80% based on these criteria. The AMAROS trial was underpowered and did not meet its primary endpoint of non-inferiority due to a very low event rate. However, the Independent Data Monitoring Committee (IDMC) approved the decision to proceed with the final analysis.

The above results (**Table 2**) reveal no statistically significant difference in clinical outcomes between the two groups in terms of axillary recurrence, disease-free survival (DFS) and overall survival (OS).

**Table 2:** AMAROS: 5 years results (5).

	ALND	Axillary RT	p value	HR
AR	0.43% (4/744)	1.19 (7/681)	NS	0.00-5.27
DFS	86.9%	82.7%	NS- 0.18	1.18 (95% CI 0.93-1.51)
OS	93.3%	92.5%	NS- 0.34	1.17 (95% CI 0.85-1.62)
Lymphedema	23%	11%	0.0001	
Arm circumference > 10%	13%	5%	0.0009	

AR, Axillary recurrence; DFS, Disease Free Survival; OS, Overall survival; HR, Hazard ratio.

Nonetheless, a notable finding was a striking difference in the incidence of lymphedema reported between the two groups with axillary RT associated with a significant reduction in rates of lymphoedema and absolute differences in arm circumference measurements (> 10%) at 5 years.

A 10-year update of the AMAROS trial was presented at the San Antonio Breast Cancer Symposium in 2018 and continued to show no differences in the aforementioned clinical endpoints. Moreover, quality-of-life scales did not differ by treatment through 5 years (6) (**Table 3**).

**Table 3:** AMAROS: 10-year update (6).

	ALND	Ax RT	p value	HR
AR	0.93% (7/744)	1.82% (11/681)		1.71 (95% CI, 0.67-4.39)
DFS	75.0% (95% CI, 71.5 to 78.2)	70.1% (95% CI, 66.2 to 73.6) i	0.11	1.19 (95% CI, 0.97-1.46)
OS	84.6% (95% CI, 81.5 to 87.1)	81.4% (95% CI, 77.9 to 84.4)	0.26	1.17 (95% CI, 0.89-1.52)
LRR	3.59%	4.07	0.69	

LRR, Loco-regional recurrence.

Interestingly, more cases of second primary tumors were observed following axillary RT (75/681), with one-quarter of these (n=21) in the contralateral breast. This compared with 57/744 cases in the ALND group, of which 11 were in the contralateral breast (p=0.035). Given the low overall numbers of second primary tumors and the relatively small differences in irradiated volumes between randomized groups, it is difficult to draw clear conclusions about causation, and a difference due to chance remains a possibility.

The OTOASOR trial was conducted around the same time (2002–2009) and had a similar trial design to AMAROS with the publication of results after eight years of follow-up (7).

This trial was a smaller single center, randomized controlled, non-inferiority study that recruited 525 patients with clinically node-negative tumors up to 3cm in size. SLN positive patients were randomized to either completion ALND or regional nodal irradiation (RNI) that encompassed all levels of axillary nodes together with the supraclavicular fossa- 25 fractions of 2 Gy.

No differences were observed between the groups with respect to axillary recurrence, DFS or OS (**Table 4**), with the caveat that the overall event rate was much lower than anticipated.

**Table 4:** Results of the OTOASOR trial (7).

	ALND	Axillary RT	p value
AR			
Total (%)	5 (2.0)	4 (1.7)	1.00
Isolated (%)	1 (0.4)	2 (0.8)	0.61
Survival at 8 years			
OS (%)	190 (77.9)	195 (84.8)	0.060
DFS (%)	176 (72.1)	178 (77.4)	0.51
Alive with recurrence (%)	14 (5.7)	17 (7.0)	
Died of breast cancer (%)	34 (13.9)	20 (8.7)	
Died of other cause (%)	20 (8.2)	15 (6.5)	
Morbidity at 1 year*	15.3%	4.7%	

*Clinical signs of lymphedema, paraesthesia, swelling, arm pain and shoulder mobility issues.

As observed in the AMAROS trial, the morbidity associated with ALND was much higher than for RNI. Combining ALND with RT for those patients with a higher nodal burden (> 4 nodes) further increased upper limb morbidity (18/57, 31.5%). However, there were not statistically significant or clinically relevant differences observed in QOL between the surgery and radiation groups.

In summary, axillary RT is an acceptable and safe alternative to ALND for clinically node negative patients with a positive SLN biopsy and otherwise similar clinical characteristics for enrolment in the AMAROS and OTOASOR trials. A key finding is the statistically and clinically significant reduction in arm morbidity for axillary RT compared to ALND.

However, there remain some outstanding clinical questions which require resolution. Firstly, there may be a cohort of patients without a high burden of axillary disease who are at lower risk and might safely be spared comprehensive axillary RT (as per AMAROS and OTOASOR protocols). There is evidence from clinical trials such as ACOSOG Z0011 and AMAROS that further axillary treatment may be unnecessary for SLN biopsy-positive patients with limited disease in sentinel nodes. It should be noted that the AMAROS trial did not include a non-intervention arm, and although axillary RT is non-inferior to ALND and associated with reduced morbidity, evidence suggests that many of these lower-risk patients may not require further definitive axillary treatment at all. Breast cancer treatments are increasingly tailored to individual patients based on disease biology and characteristics. These nuanced approaches may permit the identification of subgroups of patients who may benefit from extended irradiation with nodal fields that include the internal mammary nodes (IMN) (NCIC MA 20 and EORTC 2292) (8, 9).

### Evidence for omission of further axillary treatment in sentinel node positive patients

There are two seminal trials (IBCSG 23-01 and ACOSOG-Z0011) that have addressed the issue of whether any further axillary treatment (completion ALND/axillary RT) is necessary after primary surgery in SLN biopsy positive patients (10, 11).

The IBCSG 23-01 trial specifically included only patients with micro-metastases (≤2mm and >0.2mm) in the SLNs and these represented a very small “low risk” cohort within the AMAROS and OTOASOR trials (10). A total of 934 patients with micro-metastatic nodal disease were randomized to either completion ALND or observation only. The majority of patients within the no ALND group received whole breast radiotherapy (WBRT) as a component of breast conservation therapy. Of these, 80 patients (19%) received partial breast radiotherapy with intra-operative electron partial breast irradiation (ELIOT) that would be unlikely to capture lower axillary lymph nodes at levels 1 and 2. In addition within the no ALND group, 12 patients (3%) had wide local excision only and 42 patients (9%) underwent mastectomy and were not irradiated. No significant differences in DFS nor OS were observed after 5 years of follow-up. Results of this trial provides reassurance that definitive axillary treatment for this “low risk” group with micro-metastatic disease only can be safely omitted and thereby spare any additional morbidity.

The ACOSOG Z0011 study also examined omission of completion ALND in SLN biopsy positive patients presenting with clinically node negative EBC (11). This trial randomized 891 patients with no more than 2 positive sentinel nodes containing either macro-(>2mm) or micro-metastases to completion ALND or observation only with a slightly higher proportion of patients with micro metastases in the latter group (37.5% versus 44.8%; p=0.046). All patients underwent BCS and were scheduled to receive whole breast RT and adjuvant systemic treatment. Of note, about 50% of those patients (n=228) for whom detailed RT records were available received high tangent fields irrespective of treatment allocation (ALND 52.6%; SLN biopsy only 50%). Furthermore, 15% of patients with complete documentation received supraclavicular fossa irradiation (12). The study did not reach the pre-specified sample size of 1900 participants and closed early on the recommendation of the IDMC with only 50% accrual. This was due to the much lower than expected event rate making the estimated 500 deaths required for the study to have 90% power to confirm non-inferiority of SLNB alone compared with ALND unfeasible in terms of timescale. An updated analysis of this trial at 10 years was reassuring and showed continuing low rates of axillary relapse (0.5% in the completion axillary dissection arm and 1.5% in the SLN biopsy arm).There were no significant differences in outcomes for regional recurrence (p=0.28) with comparable 10-year overall survival for SLN biopsy alone (86.3%) and ALND (83.6) (HR 0.85; p=0.02) (13). Some patients had irradiation protocol variations that could potentially have resulted in small alteration of outcomes but are unlikely to have been responsible for equivalence of clinical outcomes and non-inferiority being maintained at 10 years follow up. The POSNOC trial has recently completed accrual and will provide information on outcomes in SLN biopsy positive patients with macro metastases in 1 – 2 nodes and not in receipt of breast irradiation (mastectomy group) (14).

## Role of regional RT in post-operative patients

Irradiation of the axillary, supraclavicular and internal mammary lymph nodes is an integral part of adjuvant therapy for some breast cancer patients. Multiple clinical trials testify to the benefits of nodal radiation therapy in improving outcomes (8, 9). Two recent trials employing field-based planning techniques with attention to quality assurance issues have demonstrated the benefit of regional nodal irradiation (RNI) when administered post-operatively in early-stage breast cancer patients. The NCIC Clinical Trials Group MA.20 trial evaluated the role of RNI (axilla, supraclavicular and internal mammary) amongst 1832 women with node-positive or high-risk node-negative breast cancer treated with breast-conserving surgery and adjuvant systemic therapy (8). Randomization was to whole breast irradiation (WBI) only or WBI combined with RNI (using modified wide tangent fields [14 – 18MV]). Addition of RNI to loco-regional treatment significantly increased relative DFS by 24%, with an absolute improvement of 5% at 10 years. The majority of regional treatment failures involved either the axillary (63%) or the supraclavicular nodes (27%) and hence the main treatment benefit related to reduction in rates of regional recurrence. There was no significant improvement in overall survival attributable to RNI (HR0.76; p=0.07) but marginal gains (~4%) in distant disease–free survival at 10 years (86.3% in RNI vs. 82.4% in the control group, p=0.03) (HR 0.64; p=0.002). It remains unclear which sites of nodal irradiation (internal mammary, supraclavicular, level III axillary, or all three) were responsible for improvements in DFS.

The EORTC 22922/10925 randomized 4004 patients with centrally or medially located primary tumors irrespective of whether the axillary node positive or negative (9). A total of 50Gy was delivered in 25 fractions with a mixed technique of 6MV photons (26Gy in 13 fractions) and 12MeV electrons (24Gy in 12 fractions) to medial supraclavicular and internal mammary node (RNI). At a median follow-up of 15.7 years, the RNI group showed a significant reduction in breast cancer mortality (HR 0.81; 95%CI 0.69–0.94; p = .005) and breast cancer recurrence (HR 0.87; 95%CI 0.77–0.98; p=0.024). A slight improvement in OS at five years was noted, which just reached statistical significance (HR 0.87; 95%CI 0.76–1.00; p=0.056). No difference was observed in the incidence of second malignancies, contralateral breast cancer, or cardiovascular deaths. A meta-analysis of the MA-20, EORTC and a French trial of RNI revealed a benefit for overall and metastases-free survival (HR 0.88; 95%CI 0.8–0.97; p=0.012) (15). Furthermore, the Danish Breast Cancer Cooperative Group (DBCG) registry reported outcomes for 3089 patients managed with a standard policy of internal mammary node (IMN) irradiation Right-sided breast cancer patients received IMN irradiation whereas this was omitted for left-sided cancers because of the risk of radiation-induced heart disease (16). The 15-year breast cancer mortality with IMN irradiation was 31.7% compared with 33.9% without (adjusted HR, 0.88 95%CI, 0.78-1.00; p = .05).

An overall comparison of the MA-20, EORTC and Danish trials in terms of their patient populations shows that the Danish study confined recruitment to axillary node-positive patients compared to the MA.20 and EORTC trials with 90% and 56% of node-positive patients, respectively. These differences in axillary nodal status likely contribute to observed differences in clinical outcomes. The latter may also relate to variations in chemotherapy schedules.

A large meta-analysis presented as an abstract in 2018 further evaluated the benefits of RNI based on individual patient data from randomized trials involving 13,404 patients in 14 studies (15). This analysis found no effect of RNI on breast cancer recurrence or mortality among 8 studies undertaken between 1961 and 1978. By contrast, in the six studies conducted after 1989, RNI significantly reduced not only breast cancer recurrence but also breast cancer-specific mortality. The risk of any death was reduced and there was no increase in non-breast cancer-related mortality (relative risk 0·88 (95% CI 0·82–0·95), p=0·002).

Absolute benefits were predictably greatest for patients with a higher number of involved axillary lymph nodes and hence nodal burden. The proportional benefits were similar across various tumor and treatment-related parameters and translated into a greater absolute benefit for those patients with a higher baseline risk for breast cancer-related events. It is reasonable to assume that as RT techniques become much more efficient and accurate, the corresponding proportional benefits for individual patients may be improved.

## Role of regional radiotherapy in the neo-adjuvant era

The optimum axillary management and role of axillary irradiation in early breast cancer patients receiving neo-adjuvant chemotherapy (NACT) remains unclear and continues to evolve. However, there is general consensus that women who are clinically node-negative (cN0) at presentation and are found to have a negative SLN biopsy after NACT do not require any further axillary treatment (17, 18). However, axillary RT (instead of ALND) may be considered for cN0 patients who are found to have fibrosis in 1 or 2 nodes and for those found to have only micro metastases or isolated tumor cells in sentinel nodes as per some guidelines (19).But there is no robust evidence to support these latter recommendations for axillary RT. The role of axillary RT in biopsy –proven cN1 patients who are down staged and have no residual tumor in sentinel lymph nodes after NACT (ypN0) is even more controversial with lack of level 1 evidence from randomized trials at the present time.

Two retrospective national cancer database (NCDB) registry-based studies explored the role of RNI in these patient cohorts who underwent breast conserving surgery. The NCDB2003–2011 study involved more than 5000 clinically node positive patients (T1-3) undergoing NACT and found no benefit in terms of overall survival from addition of RNI to breast radiotherapy (WBI) for either ypN0 or ypN+ patients (20). An updated study from the same group (NCDB 2010–2015) involving 9474 patients similarly reported no benefit from addition of RNI to WBI, regardless of pathological nodal status (21).

A retrospective study (KROG 1 6–06) from South Korea involved 261 clinically node positive (41%) and negative patients with no residual nodal tumor (ypN0) at the time of axillary surgery post-NACT. The authors found no effect of RNI on DFS or OS, irrespective of response to NACT (22). It is conceivable that the lack of any benefit from RNI in breast conservative therapy patients might be attributed to the use of tangential beams in many of these series which can capture up to 80% of nodes at levels I and II of the axilla.

NACT results in a complete pathological response in the axillary nodes in 40-70% of patients depending on tumor phenotype. This intuitively questions the benefit of any further axillary treatment in this ypN0 group of patients (23). Current evidence suggests that core biopsy-proven node positive women with normalization of axillary nodes post-NACT can be safely considered for sentinel node biopsy alone provided at least 3 nodes are removed and dual mapping techniques employed. Alternatively, the biopsied node can be clipped and retrieved at the time of SLN biopsy – the so-called targeted ALND (24, 25). The ongoing NSABP B-51/RTOG 1304 and ATNEC trials will examine the role of RNI in ypN0 patients (26, 27). The Dutch RAPCHEM;BOOG 2020-03 registry study is the first prospective evidence for the safety of selective de-escalation of radiation therapy after NACT for biopsy-proven clinically node positive T1-2N1 early breast cancers. Patients were grouped into low, intermediate and high risk for recurrence based on pre-defined criteria and all underwent ALND post-NACT. Radiation therapy (local and/or regional) was omitted for ypN0 patients and those with residual nodal disease (ypN1) received irradiation of local tissues only. Overall 5 year rates of loco-regional recurrence were only 2.2% with this policy of response-adapted radiotherapy (28).

## Concluding comments

There are several ongoing trials that aim to address some key outstanding questions in relation to the role of radiation therapy in management of early breast cancer patients. The POSNOC (POsitive Sentinel NOde: adjuvant therapy alone versus adjuvant therapy plus Clearance or axillary radiotherapy) trial is a predominantly UK-based multicenter trial that notably includes women undergoing primary surgery with either mastectomy or breast conserving therapy in conjunction with SLN biopsy (14). Women with 1 or 2 sentinel nodes containing macro metastases are eligible for randomization as well as those with extra-nodal invasion. This trial will determine whether SLN biopsy alone yields equivalent clinical outcomes to further axillary treatment (completion ALND or axillary irradiation) POSNOC has an in-built radiotherapy quality assurance programme coordinated by the UK Radiotherapy Trials Quality Assurance Group. The NSABP B-51/RTOG 1304 study will examine whether post-mastectomy radiotherapy combined with RNI or addition of RNI to breast RT following breast conserving surgery increases invasive breast cancer recurrence-free interval in cN1 patients converting to ypN0 after NACT (26). The ATNEC trial will randomize ypN0 patients to either observation only following SLN biopsy or further axillary treatment (completion ALND or axillary RT) (27). Other de-escalation studies include the ALLIANCE A011202 trial that will determine whether axillary RT combined with RNI is non-inferior to completion ALND and RNI for higher risk patients with residual nodal macro metastases after NACT followed by SLN biopsy (29). The international TAXIS (SAKK 23–16/IBCSG 57–18/ABCSG-53/GBG 101) trial has a complex study design that recruits a mixed population of clinically node positive patients undergoing NACT or upfront surgery. The trial aims to test the hypothesis that axillary RT in pathologically node-positive patients is non-inferior to ALND in terms of DFS (30). The trial incorporates methods for tailored axillary surgery such as targeted ALND. Finally, a unique feasibility study, entitled the NEONOD2 trial (NCT040196780) will evaluate the safety of omitting both ALND and RNI in patients with micro metastatic disease only in sentinel nodes (31).

## Author contributions

JB is the corresponding author. TE and UJ are joint first authors. All authors contributed to the article and approved the submitted version.
